# Variation in Host Resistance to Blastomyces dermatitidis: Potential Use of Genetic Reference Panels and Advances in Immunophenotyping of Diverse Mouse Strains

**DOI:** 10.1128/mbio.03400-21

**Published:** 2022-01-04

**Authors:** Elaine M. Kohn, Cleison Taira, Hanah Dobson, Lucas Dos Santos Dias, Uju Okaa, Darin L. Wiesner, Marcel Wüthrich, Bruce S. Klein

**Affiliations:** a Department of Pediatrics, University of Wisconsin School of Medicine and Public Health, University of Wisconsin—Madison, Madison, Wisconsin, USA; b Department of Internal Medicine, University of Wisconsin School of Medicine and Public Health, University of Wisconsin—Madison, Madison, Wisconsin, USA; c Department of Medical Microbiology and Immunology, University of Wisconsin School of Medicine and Public Health, University of Wisconsin—Madison, Madison, Wisconsin, USA; Albert Einstein College of Medicine

**Keywords:** fungi, immunity, resistance, mice, blastomycosis, adaptive immunity

## Abstract

Host genetic determinants that underpin variation in susceptibility to systemic fungal infection are poorly understood. Genes responsible for complex traits can be identified by correlating variation in phenotype with allele in founder strains of wild mice with known genetic variation, assembled in genetic reference panels. In this work, we describe wide natural variation in both primary and acquired resistance to experimental pulmonary blastomycosis in eight founder strains, including 129, A/J, BL/6, CAST, NOD, NZO, PWK, and WSB of the Collaborative Cross collection, and the inbred DBA strain. These differences in susceptibility across strains were accompanied by sharp differences in the accumulation and function of immune cells in the lungs. Immune perturbations were mapped by identifying reagents that phenotypically mark immune cell populations in the distinct strains of mice. In particular, we uncovered marked differences between BL/6 and DBA/2 mouse strains in the development of acquired resistance. Our findings highlight the potential value in using genetic reference panels of mice, and particularly the BXD (recombinant inbred strains of mice from a cross of C57BL/6J and DBA/2J mice) collection harboring a cross between resistant BL/6 and susceptible DBA/2 mice, for unveiling genes linked with host resistance to fungal infection.

## INTRODUCTION

Laboratory mice offer power in understanding the complex genetic bases of human disease through translational research. Inbred mouse strains can be crossed to create genetic reference panels, which when inbred into unique strains are called recombinant inbred (RI) mice and have fixed, homozygous alleles that vary from strain to strain. Genetic reference panels allow the investigation of complex trait etiology through the integration of phenotypic observations with known genetic variation (1). The stabilization of known allelic variation in mouse populations provides a basis for phenotypic data integration and subsequent genetic causal analysis via high-resolution quantitative trait locus (QTL) mapping (1, 2).

The Collaborative Cross (CC) is a genetic reference panel of RI mice derived from eight founder strains: 129S1/SvlmJ, A/J, C57BL/6, NOD/ShiLtJ, NZO/HiLtJ, CAST/EiJ, PWK/PhJ, and WSB/EiJ. These founder strains naturally harbor 89% of the known genetic diversity in mice (3). Much of the genetic variation is provided by the three wild-derived strains CAST, PWK, and WSB as they each originate from separate subspecies (Mus musculus *castaneus*, M. musculus *musculus*, and M. musculus *domesticus*, respectively), as opposed to the five laboratory strains, which are derived largely from M. musculus *domesticus* alone (4).

The CC has already been used to identify immunological traits (5, 6) in the context of infection models (7–9), including in that of primary infection with several mycoses. CC strains in the process of development (“pre-CC,” at ∼80% homozygosity) were used to identify genetic susceptibility loci in an aspergillosis model (10). Other investigations used several RI panels, including one derived from BL/6 and DBA/2 mice alone, called the BXD, to identify loci associated with complex disease traits in a Candida albicans model, which implicated a complement gene with a loss-of-function mutation (11), as well as several novel loci (12). In another study (13), investigators used BXD mice to confirm that susceptibility to *Coccidioides* sp. in C57BL/6 mice is associated with the expression of a truncated splice variant of Dectin-1 (Clec7a). Genetic variation in mice clearly plays a role in their primary immunity to these and other pathogens.

In humans, host-level variation is thought to affect disease susceptibility and acquired resistance to fungal infection. Endemic systemic mycoses, including blastomycosis, histoplasmosis, and coccidioidomycosis, demonstrate racial and ethnic variation in infection or disseminated disease. Blastomyces dermatitidis, the agent of blastomycosis, infects apparently immunocompetent hosts (14). In Wisconsin, people of Hmong ancestry have a higher incidence of blastomycosis (168 versus 13/100,000 for people of European ancestry) (15). A study of a 2010 outbreak excluded common risk factors involving environmental exposure to the fungus (15). Genome sequencing of Hmong patients led to the identification of a susceptibility locus: interleukin-6 (IL-6). A deficit in IL-6 production discovered in cells from Hmong donors was linked to Hmong susceptibility (16). Other genetic sources of impaired immunity remain unknown. Due to the development of murine genetic reference panels that include variation from three wild-derived strains, we now have tools to uncover complex susceptibility and resistance loci and discover the mechanisms responsible for both primary and acquired immunity to this disease.

We investigated susceptibility to blastomycosis across diverse mice with the long-term goals of identifying causal genetic factors. We wondered to what degree mice naturally vary in their resistance. Here, we demonstrate marked phenotypic variation in both primary and acquired immunity to blastomycosis across genetically diverse strains of mice. We establish a proof of concept for the use of the Collaborative Cross genetic reference panels for the investigation of genetic sources of host resistance. In addition, we uncover marked differences in the development of acquired resistance between BL/6 and DBA/2 mice, paving the way toward utilizing the CC, Diversity Outbred (DO), or BXD (recombinant inbred strains of mice resulting from a cross of C57BL/6J and DBA/2J mice) genetic reference panels for identifying genes associated with acquired resistance.

## RESULTS

### Diverse mice differ in early responses to primary fungal pulmonary infection.

**FIG 1:**
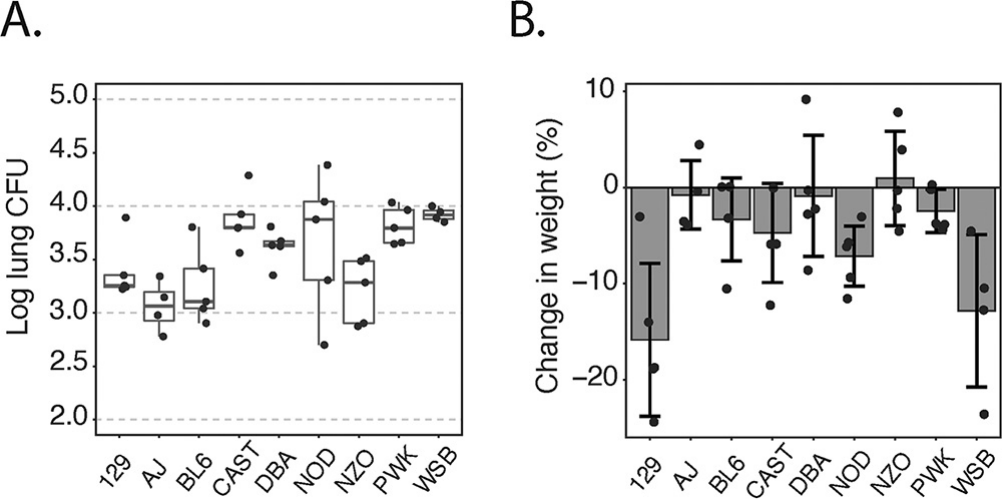
Protection against primary fungal infection varies across CC founder strains. Mice were challenged with 2 × 10^4^ cells of B. dermatitidis yeast strain ATCC 26199 (*n* = 4 to 5 mice/group). (A) Fungal burden at day 2 p.i. (*P* = 0.0015). Significance was determined by type III ANOVA of log_10_-transformed CFU. (B) Weight change over 2 days of infection (*P* = 0.0006). Significance was determined by type III ANOVA. Data are representative of results from two independent experiments.

We first sought to assess the susceptibility of the eight CC founders and DBA mice to primary pulmonary blastomycosis. Mice were infected intratracheally (i.t.) with 20,000 cells of B. dermatitidis virulent yeast strain ATCC 26199. At 48 h postinfection (p.i.), the mice were euthanized and assessed for both fungal burdens and weight loss over the course of the infection. We observed significant variation in fungal burdens at this early time point, with about a log spread (*P* = 0.0015 by regression analysis of log_10_-transformed CFU) (Fig. 1A). CAST, PWK, and WSB mice had higher CFU than some other strains. DBA and NOD mice had high CFU as well, although NOD mice had a sizeable spread. In addition to fungal burdens, founder strains showed a wide range of weight loss (*P* < 0.001) (Fig. 1B). 129 mice showed significant wasting, losing an average of 15% of their body weight at day 2 p.i., despite having a relatively moderate fungal burden. WSB mice had considerable wasting as well, averaging 13%. Other strains (A/J, DBA, and NZO) had no weight loss. The variation in primary resistance so soon after infection implies a strong genetic contribution to the innate immune responses.

### Acquired resistance against fungal infection varies across diverse strains of mice.

**FIG 2:**
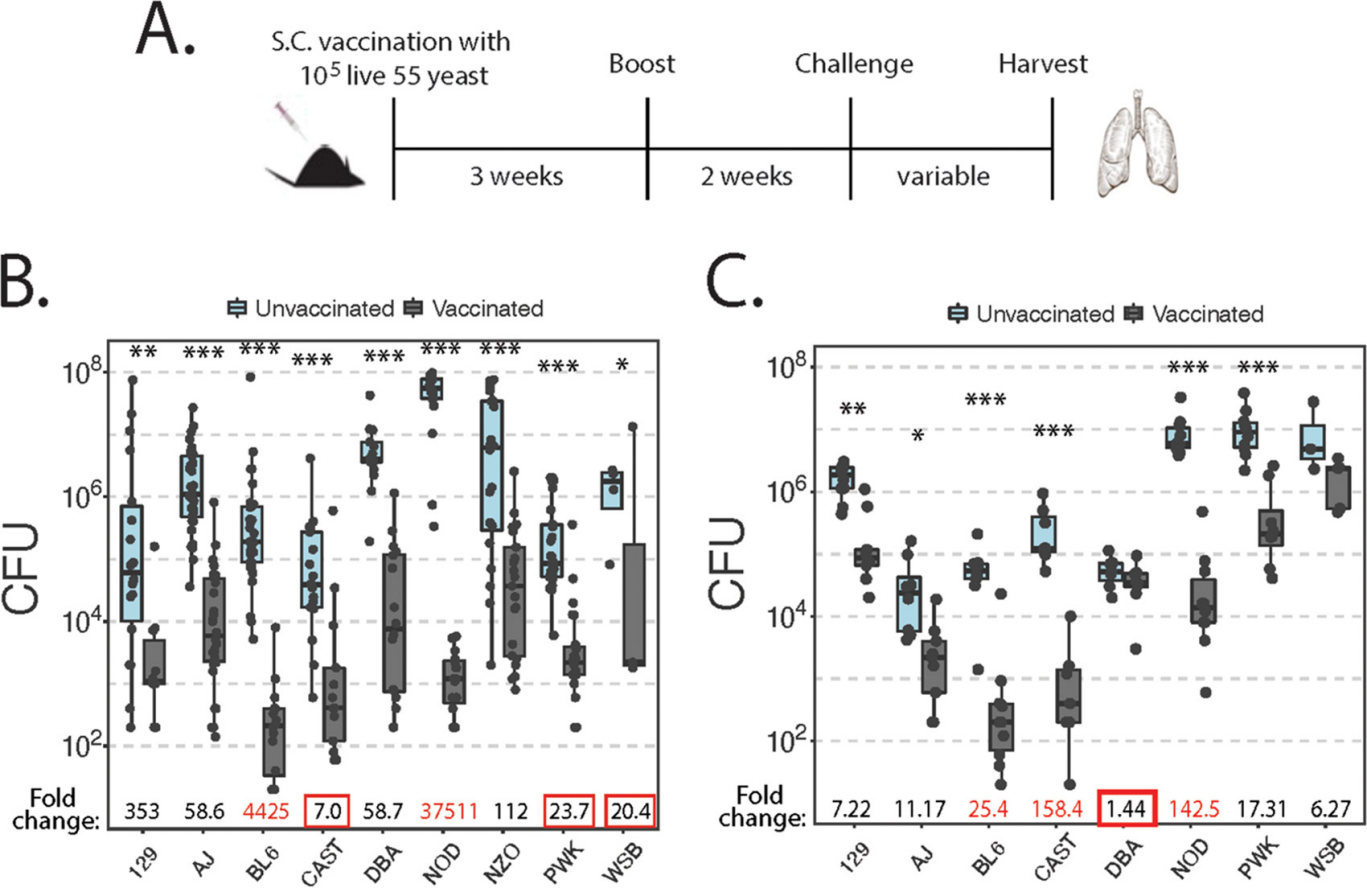
Vaccine-induced protection against fungal infection varies across the CC founder strains. (A) Experimental model. Vaccinated mice were given 10^5^ live attenuated B. dermatitidis yeast strain 55 cells subcutaneously and boosted before challenge. Vaccinated and unvaccinated mice were challenged with cells of virulent B. dermatitidis yeast strain ATCC 26199 i.t. and harvested when moribund. (B) Fungal burden at day 14 p.i. when mice were challenged with 10^3^ yeast cells. Vaccine-induced protection significantly differs across the strains (interaction coefficient *P* < 0.0001 by two-way ANOVA). Red numbers highlight especially high fold changes, and boxes highlight especially low fold changes (*n* = 3 to 33 mice/group; mean and median, *n* = 22). (C) Fungal burden at day 9 p.i. when mice were challenged with 2 × 10^4^ yeast cells. Vaccine-induced protection significantly differs (*P* < 0.0001). Red numbers highlight especially high fold changes, and boxes highlight especially low fold changes (*n* = 3 to 10 mice/group; mean and median, *n* = 8).

To assess acquired resistance to fungal infection, we subcutaneously (s.c.) vaccinated mice with live attenuated B. dermatitidis yeast cells as previously described (17, 18). Mice were boosted 3 weeks later and then rested for 2 weeks before pulmonary challenge with virulent yeast and subsequent lung harvest (Fig. 2A).

Some of the founder strain mice are quite small (e.g., CAST and WSB), with some mice weighing less than 10 g. We were concerned that a high inoculum would overwhelm the small mice, so we challenged all strains with a noncustomary small inoculum of 3 × 10^3^ virulent yeast cells. We then harvested animals when the first unvaccinated control groups became moribund, which was about day 14 p.i. (Fig. 2B). Small mice did not appear to become overwhelmed with fungal burdens, and larger mice, such as DBA, NOD, and NZO mice, all had higher CFU on average than the small mice.

The founder strains differed in susceptibility to primary infection (e.g., unvaccinated control), with about a 4-log spread, as seen in Fig. 1A (*P* < 0.0001). In addition, CC founder strains demonstrated a wide spread in acquired resistance (interaction coefficient *P* < 0.0001), although all strains had a significant reduction in the fungal burden. The reduction in CFU upon vaccination ranged from 7-fold in CAST mice to over 37,000-fold in NOD mice. The striking resistance in the NOD strain stems in part from its high susceptibility to primary infection; e.g., unvaccinated NOD control mice had the highest mean burden at nearly 10^8^ CFU. Although we observed significant variation in acquired resistance among the strains, the low inoculum size resulted in a considerable spread of fungal burdens within groups (1 to 5 logs). For this reason, we exclusively used a customary challenge inoculum (for this model) of 2 × 10^4^ virulent yeast cells in subsequent experiments.

When challenged with 2 × 10^4^ virulent yeast cells, the first unvaccinated strains became moribund at day 9 p.i. Founder strains maintained the variation in acquired resistance observed with the smaller inoculum (Fig. 2C) (interaction coefficient *P* < 0.0001). However, the spread of CFU within individual strains fell dramatically using a higher inoculum (to 1 to 2 logs within groups). Unvaccinated strains again differed in susceptibility to primary infection, with about a 3-log spread (*P* < 0.0001). CAST, NOD, and BL/6 mice showed excellent protection, with fold changes of 158.4, 142.5, and 25.4, respectively. Strikingly, DBA mice showed a severe deficit in acquired protection, with a fold reduction in CFU of only 1.44.

We investigated the contribution of gender to susceptibility. We saw highly variable, inconsistent effects of gender across many experiments. Although there is often an overall effect of sex, when strains were combined, there was no interaction of sex with our experimental factors of interest, e.g., founder strain and vaccination status. Therefore, we included both male and female mice for every strain in subsequent experiments and averaged the results on the levels of strain and vaccination condition.

### Validation of immunophenotyping by flow cytometry.

We next sought to assess cellular responses to infection across the CC founder strains. We first needed to establish reagents that reliably identify phenotypic markers in every strain because different strains of mice carry natural allelic variations that result in protein polymorphisms. Due to the extreme specificity of monoclonal antibodies, not all commercially available mouse antibodies stain every phenotypic marker in every strain. Without reliable reagents, it can thus be unclear whether the observed cellular deficits are genuine or technical artifacts.

**FIG 3:**
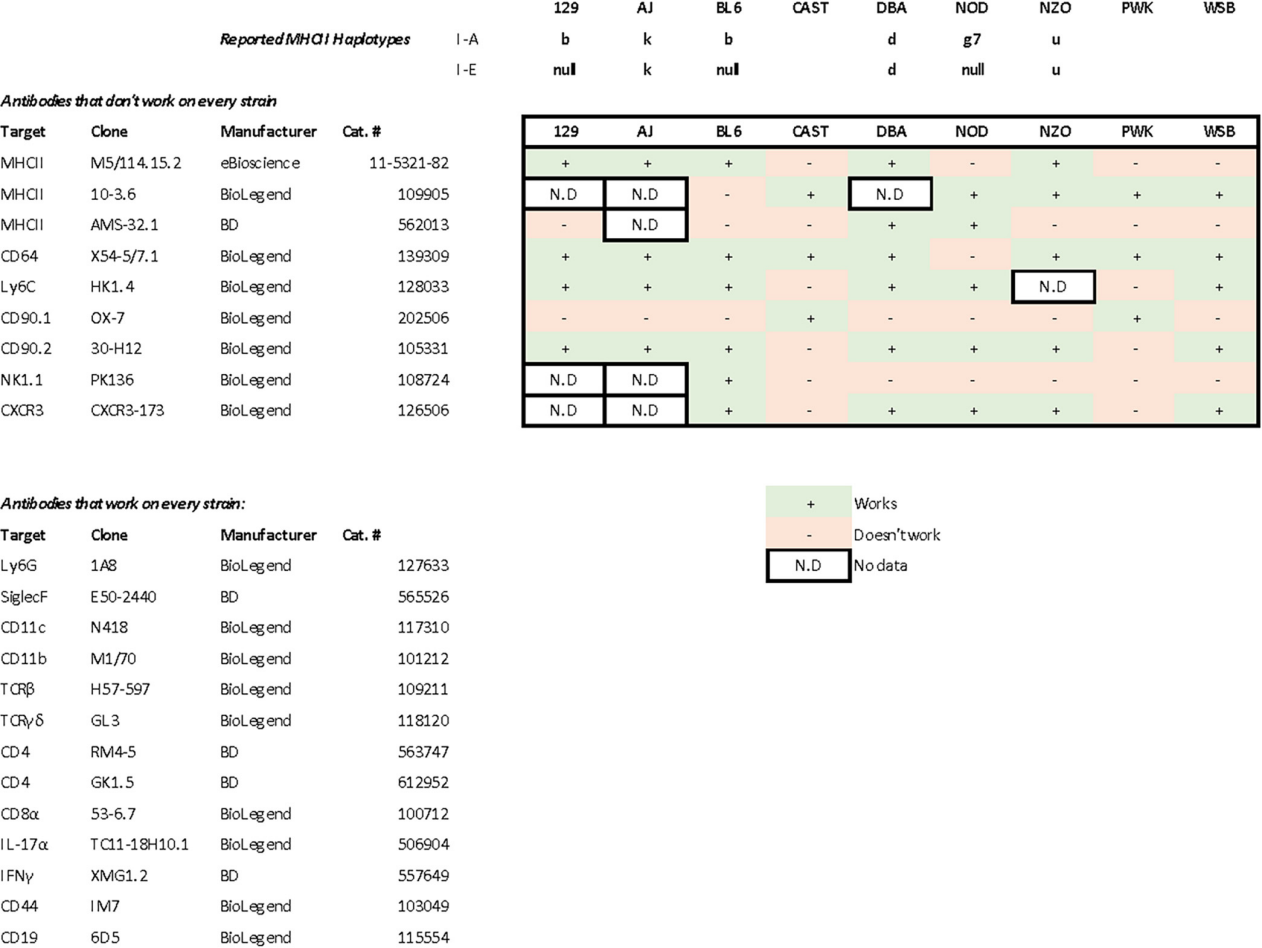
Summary of the staining efficiencies of various antibody clones in distinct mouse strains. Isolated lung cells or splenocyte aliquots were stained with various antibodies as described in Materials and Methods and analyzed via flow cytometry. Data are representative of results from at least two experiments. TCRβ, T cell receptor β.

Some markers, such as major histocompatibility complex class II (MHCII), vary widely in known haplotypes. The wild-derived strains CAST, PWK, and WSB lack definitive identification of MHCII haplotypes despite genome sequencing (19) and assembly by the Mouse Genomes Project (https://www.sanger.ac.uk/data/mouse-genomes-project/). While there is no available information on WSB MHC haplotyping, CAST mice are suggested to be MHC H2 null (20), and PWK mice are suggested to have a novel haplotype (21). We found that MHCII antibody clones M5/114.14.2 and 10-3.6 together stain MHCII in all the founder strains (Fig. 3; see also Fig. S1 in the supplemental material).

10.1128/mbio.03400-21.1 FIG S1 MHCII antibody validation. Flow cytometry analysis of APCs was performed using several antibody clones, all reported to stain multiple haplotypes. (A) MHCII staining of lung cells with antibody clone M5/114.15.2. (B) MHCII staining with clones AMS-32.1 and 10-3.6. A single BL/6 sample was stained with the M5/114.15.2 clone as a positive control. Download FIG S1, TIF file, 0.8 MB. Copyright © 2022 Kohn et al. 2022 Kohn et al. https://creativecommons.org/licenses/by/4.0/ This content is distributed under the terms of the Creative Commons Attribution 4.0 International license.

To enable our cellular analyses, we rigorously tested every antibody used in our flow cytometry analysis (Fig. 3; Fig. S2). We sought staining properties comparable to those observed in BL/6 mice. We report antibody clones that do or do not work on a specific founder strain to facilitate future immunophenotyping of these diverse mice. We identified an antibody cocktail that detects every marker of interest for every mouse strain, with one exception: we found no clone of anti-CD64 that labeled NOD mice. Therefore, we lack precise data for monocyte-derived dendritic cells in NOD mice; their monocyte counts were derived from total MHCII^−^ CD11b^+^ cells, and their alveolar macrophage counts were derived from total SiglecF^+^ CD11c^+^ cells.

10.1128/mbio.03400-21.2 FIG S2 Antibody staining. Isolated lung cells or splenocytes were stained with various antibodies as described in Materials and Methods. Percentages are of the parent gate. (A) Staining with CD11b (M1/70) and Ly6C (HK1.4). As Ly6C did not stain every strain, we moved to identifying monocytes as MHCII^−^ CD11b^+^ CD64^+^. (B) Staining with TCRβ (H57-597), TCRγδ (GL3), and CD90.2 (OX-7). TCRγδ^+^ cells were not observed in CAST, PWK, and WSB mice, likely due to extremely low cell counts. (C) Staining with CD90.1 (OX-7), CD90.2 (30-H12), CD19 (6D5), CD8 (53-6.7), CD4 (GK1.5), SiglecF (E50-2440), and Ly6G (1A8). (D) Staining with CD11c (N418) and CD64 (X54-5/7.1). Download FIG S2, TIF file, 3.0 MB. Copyright © 2022 Kohn et al. 2022 Kohn et al. https://creativecommons.org/licenses/by/4.0/ This content is distributed under the terms of the Creative Commons Attribution 4.0 International license.

### Diverse strains differ in both primary and acquired cellular responses to fungal infection.

**FIG 4:**
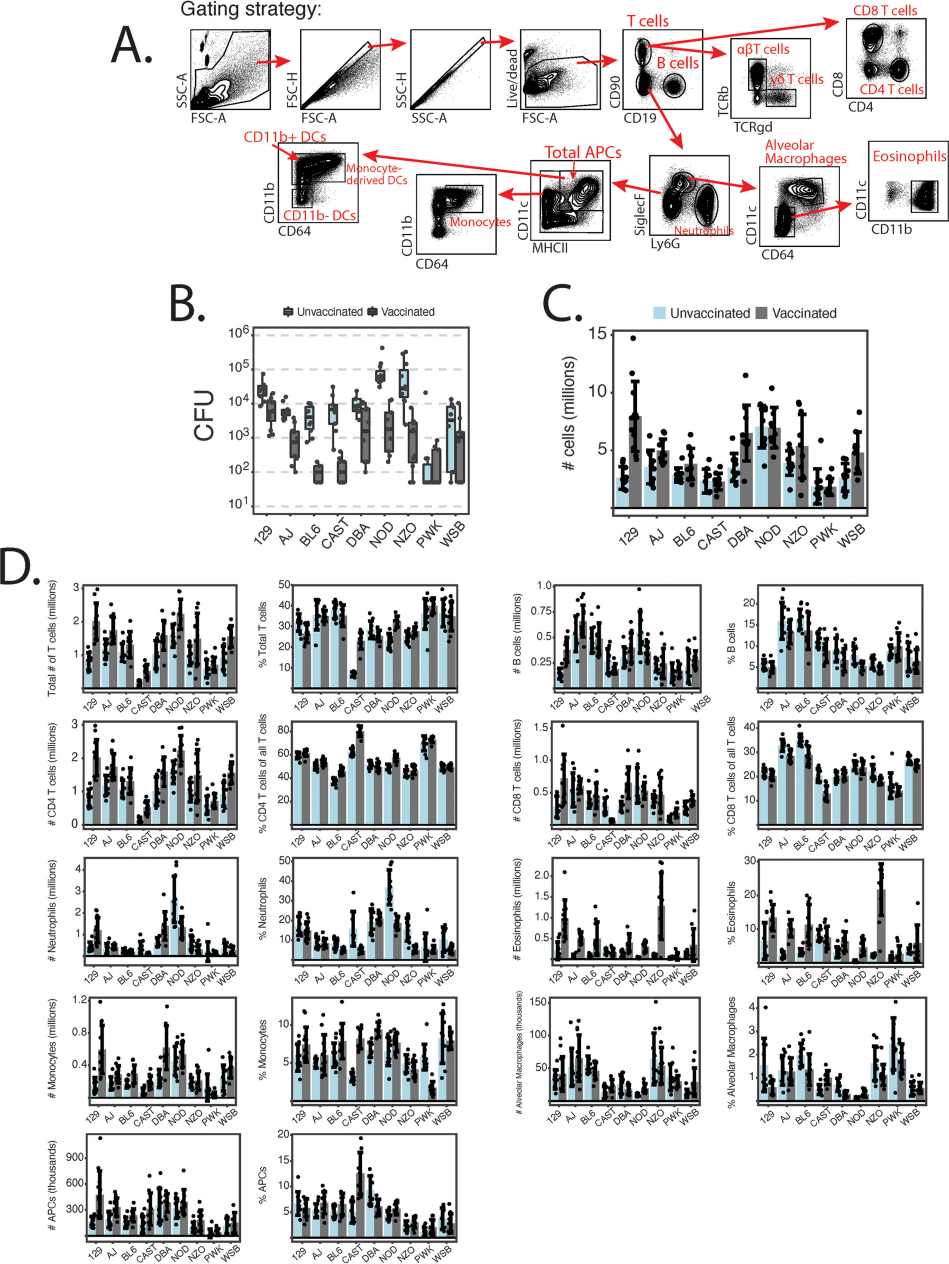
CC founder strains differ in innate and acquired cellular responses to fungal infection. Lungs of vaccinated and unvaccinated mice were harvested 4 days after infection with 2 × 10^4^ yeast cells (*n* = 9 to 12 mice/group). (A) Dual myeloid-lymphoid gating strategy used for phenotyping. SSC, side scatter; FSC, forward scatter; DCs, dendritic cells. (B) Fungal burden at day 4 p.i. Strains differ significantly when unvaccinated (*P* < 0.0001) and vaccinated (*P* < 0.0001). Zero values were set to the detection limit (50 colonies). (C) Total numbers of live cells collected from each mouse. There is a significant interaction between cellular response and mouse strain in vaccinated animals (*P* < 0.0001). (D) Counts of myeloid and lymphoid cells and their relative percentages of total live cells. Statistics were analyzed by multivariate ANOVA and Tukey’s HSD test. Data are representative of results from two experiments.

The CC founders have known diversity in resting immune phenotypes (6). We sought to assess immune phenotypes in the context of both primary infection and acquired resistance to pulmonary blastomycosis. We challenged unvaccinated and vaccinated mice and harvested lungs at day 4 p.i. to assess innate and acquired cellular responses. We used a dual myeloid and lymphoid cell gating strategy that can identify up to 14 unique cell populations per sample (Fig. 4A) (22). At day 4 p.i., the CC founder strains varied in both their innate and acquired cellular responses to infection albeit in a setting of a 3-log spread in the lung CFU across strains (Fig. 4B). The total numbers of live cells in the lungs of mice differed among strains and depended on vaccine status, mouse strain, and the interaction of the two factors (Fig. 4C) (all three *P* values were <0.0001). Because cell numbers may be confounded by baseline differences in total cell counts, we analyzed cellular responses by both the absolute number and relative percentage of the entire live-cell population collected.

Mice showed broad phenotypic diversity in cellular responses at day 4 p.i. (Fig. 4D). First, the founder strains differed in both the number and frequency of innate lung-resident alveolar macrophages and total antigen-presenting cells (APCs) (identified as MHCII^+^ CD11c^+^ from the CD90^−^ CD19^−^ SiglecF^−^ Ly6G^−^ population). While there is no vaccine effect on the number of alveolar macrophages, most strains upregulate APCs upon vaccination. The degree to which vaccination affects APC counts differs across the strains, which implies a genetic basis for the acquired responses.

Cell populations responsible for the primary response to infection—neutrophils, monocytes, and eosinophils—differ widely across the founders. The effect of vaccination on all these populations is strain dependent, again suggesting an important role for genetics.

Lymphocytes also differed in both innate and acquired responses across the strains. Unvaccinated mice differed in the number and frequency of total B and T cells as well as in CD4 and CD8 T cell numbers and their frequency among CD4 cells. Strains also differed in the way in which vaccination affected lymphocyte counts: all T and B cell phenotypes showed interaction effects between both strain and vaccination, again suggesting a strong relationship between acquired immunity and genetic background.

We were concerned that the differences in the burdens of infection observed here may confound the cellular analysis. For example, the lung burden in unvaccinated mice differed across the strains (*P* < 0.0001), as described above. Acquired resistance varied as well, with fold CFU reductions ranging from 63.4 in the CAST strain to only 2.5 in the WSB strain (*P* < 0.0001). We therefore repeated this experiment except that we recalled immune responses by challenging vaccinated mice with heat-killed yeast rather than live, virulent yeast. In this experiment, most trends observed in the regression analysis of the live challenge experiment were recapitulated (Fig. S3). Therefore, genetic background appears to strongly affect both primary and acquired cellular responses in mice in the context of pulmonary blastomycosis.

10.1128/mbio.03400-21.3 FIG S3 Immunophenotyping after challenge with heat-killed yeast. Counts of myeloid and lymphoid cells and their relative percentages of total live cells are shown. Male and female vaccinated and unvaccinated mice of each strain were included (total *n* = 220), so the harvest was split into two harvest days. There were significant batch effects between the days, so “harvest” is added as a predictor factor in the multivariate regression analysis instead of combining the two data sets. Statistics were analyzed by multivariate type III ANOVA and Tukey’s HSD test (*n* = 9 to 20 mice/strain). Download FIG S3, TIF file, 0.7 MB. Copyright © 2022 Kohn et al. 2022 Kohn et al. https://creativecommons.org/licenses/by/4.0/ This content is distributed under the terms of the Creative Commons Attribution 4.0 International license.

### Diverse strains of mice vary in the production of protective cytokines upon fungal infection.

**FIG 5:**
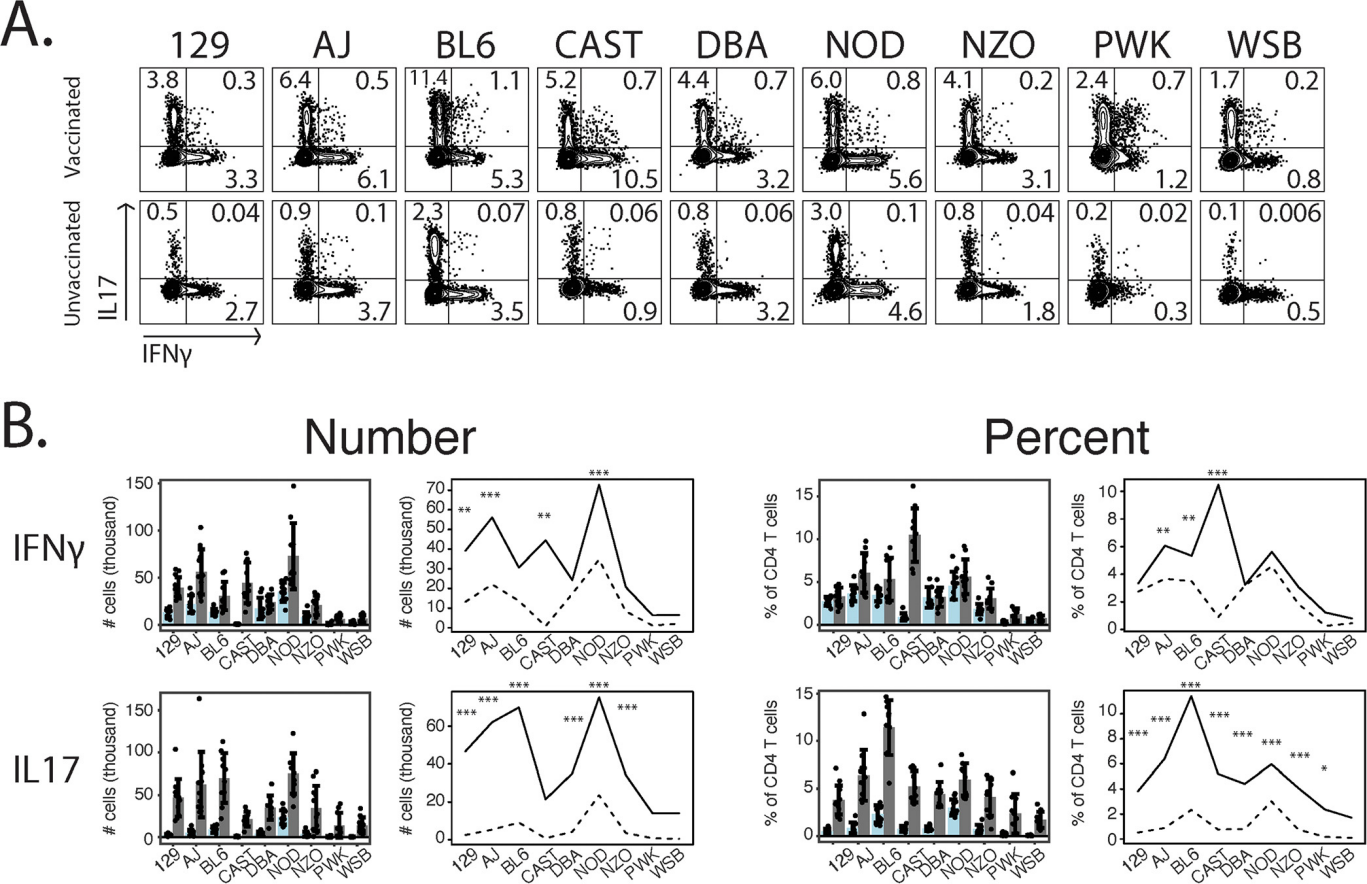
CC founders vary in cytokine production upon fungal infection. (A) Flow cytometry analysis of cytokine-producing lung T cells 4 days after infection with 2 × 10^4^ yeast cells (*n* = 9 to 12 mice/group). Numbers indicate the average percentages of cytokine-positive cells of the previous gate for the group. Plots are representative concatenates. Previous gates were live, CD19^−^ CD90^+^ CD4^+^ CD44^hi^. (B) Quantification of cytokine-producing cells. Bar graphs display means with error bars (±1 standard deviation). Interaction plots show the means for each group. Data are representative of results from two experiments.

We analyzed products made by CD4 T cells, as these T cells are mainly responsible for acquired resistance to B. dermatitidis and related fungi (23) (see CD8 T cell data in Fig. S4). Both Th1 and Th17 cells confer acquired resistance to fungi and thus serve as functional markers of resistance (24, 25). We found significant variation across the CC founders in both the frequencies (Fig. 5A) and numbers (Fig. 5B) of activated (CD44^hi^) interferon gamma-positive (IFN-γ^+^) and IL-17^+^ CD4^+^ T cells.

10.1128/mbio.03400-21.4 FIG S4 CD8 T cell functional responses vary across genotypes. (A) Flow cytometry analysis of IFN-γ^+^ and IL-17^+^ lung CD8 T cells of vaccinated and unvaccinated mice at day 4 p.i. (*n* = 9 to 12 mice/group). (B) Quantification of CD8^+^ cytokine-positive cells. Bar graphs display means with error bars (±1 standard deviation). Interaction plots show the means for each group. Download FIG S4, TIF file, 0.8 MB. Copyright © 2022 Kohn et al. 2022 Kohn et al. https://creativecommons.org/licenses/by/4.0/ This content is distributed under the terms of the Creative Commons Attribution 4.0 International license.

To better visualize the differing levels of acquired resistance across the strains, we created interaction plots alongside bar plots (Fig. 5B). The means for each strain are plotted in Fig. 5B, and lines connect either the vaccinated (solid lines) or unvaccinated (dashed lines) groups. The interactions of the strain and vaccination factor are illustrated by the different slopes of the lines. An interaction effect is evident when the shape of the vaccinated line does not mirror that of the unvaccinated line across all genotypes. Some strains demonstrated a dramatic upregulation of cytokine-positive T cells after vaccination. Vaccinated 129, A/J, and NOD mice greatly increased the numbers of both IL-17^+^ and IFN-γ^+^ cells. For 129 and NOD mice, the frequency of the IFN-γ^+^ population among total CD4 T cells did not increase to the same degree, indicating that this upregulation results from T cell influx and not increased effector function. On the other hand, their IL-17^+^ cell frequency increased substantially, indicating a real difference between the strains in acquired resistance. All strains except for WSB increased the frequency of IL-17^+^ cells among CD4 T cells. Some strains, like DBA, NZO, PWK, and WSB mice, showed no increase in the number of IFN-γ^+^ cells and much less of an increase in the number of IL-17^+^ cells than other strains, suggesting genetic deficits in recalling effector T cell responses.

### Sharp differences between BL/6 and DBA strains in acquired resistance to blastomycosis.

**FIG 6:**
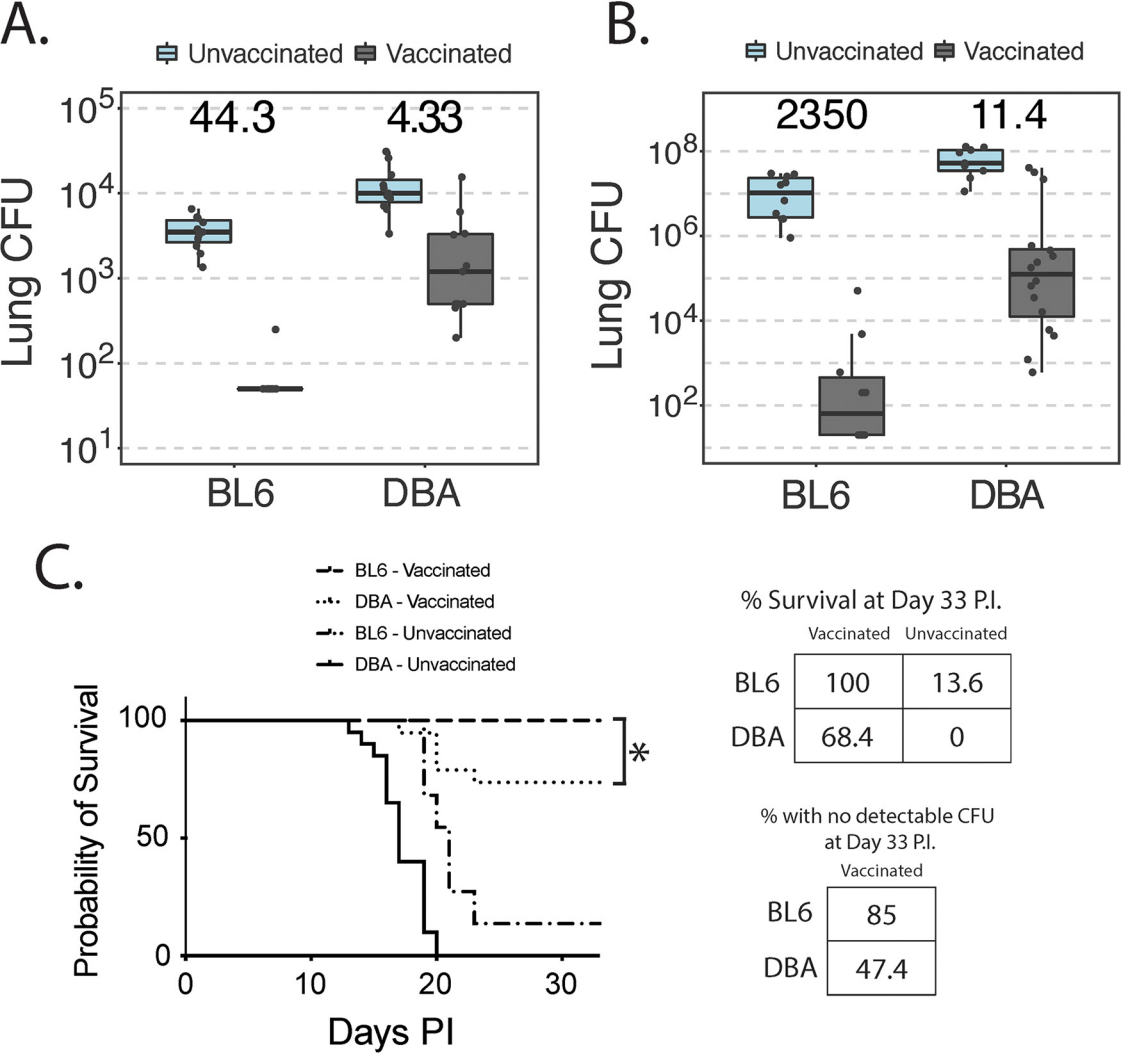
BL/6 and DBA mice differ in resistance to infection. (A) Fungal burden at day 4 p.i. Data are representative of results from two independent experiments (*n* = 4 to 6 mice/group). Zero values were set to the detection limit (50 colonies). (B) Fungal burdens in vaccinated and unvaccinated DBA and BL/6 mice at day 14 p.i. (*n* = 8 to 16 mice/group). Zero values were set to the detection limit (20 colonies). (C) Kaplan-Meier survival curve (*n* = 19 to 22 mice/group). Overall, the curves are statistically different (*P* < 0.0001). Vaccinated BL/6 mice survive significantly better than vaccinated DBA mice (*P* = 0.0151). Differences were determined by a Mantel-Cox rank test. All mice in these experiments received an inoculum of 2 × 10^4^ yeast cells.

Thus far, DBA mice demonstrated a deficit in acquired immune phenotypes relative to BL/6 mice. We sought to investigate this further and found that BL/6 mice acquire significantly better resistance to lung infection than DBA mice. Early, by day 4 p.i., vaccinated BL/6 mice showed an ∼10-times-better CFU reduction than vaccinated DBA mice (Fig. 6A) (interaction coefficient *P* < 0.001). Likewise, at day 14 p.i., when the first unvaccinated control mice became moribund and groups were sacrificed, the differences between the two strains were even more pronounced (Fig. 6B). Here, BL/6 mice demonstrated a 205-fold-better CFU reduction than that seen in the DBA mice (*P* = 0.004). The difference in acquired resistance between the strains occurred in a setting where DBA mice were slightly more susceptible to primary infection (e.g., unvaccinated mice) than BL/6 mice (*P* < 0.001 at day 4 and *P* = 0.004 at day 14). The relative difference in burdens between the two strains (about 5-fold) remained the same between days 4 and 14 p.i. in unvaccinated mice, suggesting that the even greater difference between the strains in acquired resistance at day 14 was not simply the result of greater relative differences between the unvaccinated strains at this time.

We also investigated survival after infection in these two strains of mice. We observed deficits in innate and acquired resistance to infection in the DBA mice. The naive mice succumbed to primary infection significantly earlier than did BL/6 mice (Fig. 6C). Following vaccination, DBA mice likewise had significantly higher mortality rates than BL/6 mice (*P* = 0.015). Only 68.4% of vaccinated DBA mice survived to 1 month postinfection, compared to 100% of BL/6 mice. We analyzed the burden of infection in vaccinated survivors. Surviving vaccinated BL/6 mice cleared the fungal burden from their lungs much better than DBA mice, with 85% of BL/6 mice showing sterilizing immunity, compared to 47.4% of DBA mice (detection limit = 20 colonies). Thus, this survival model underscores the observation that DBA mice have clear deficits in immunity to blastomycosis relative to BL/6 mice.

## DISCUSSION

Although some variation in primary fungal immune phenotypes has been described in diverse mice, we now have the means to identify their causal genetic factors through the use of genetic reference panels. In this work, we have identified significant phenotypic variation in both innate and acquired susceptibility to blastomycosis associated with inbred mouse strains.

Genetic reference panels of recombinant inbred mice can be used to study host-level genetic sources of disease susceptibility via the integration of phenotypic and genetic data. The Collaborative Cross was conceived nearly 2 decades ago with the ambitious goal of generating 1,000 unique CC strains to facilitate submegabase mapping resolution (26). A major limitation of the CC today is the low number of extant strains due to infertility and attrition over time (27), which limits the current mapping resolution (1). Despite limitations, the CC is the only known genetic reference panel to maintain consistent genetic variation across the entire genome, which is required for systems analysis and models human genetics well (3). On the other hand, the use of the Diversity Outbred (DO) panel offers nearly infinite heterozygosity, which allows efficient breeding and grows more robust with time (28). The major drawback of the DO panel is the necessity to genotype and haplotype reconstruct each mouse, which is made challenging by their complex genomics and unbalanced pedigree (1, 28).

Variation in fungal disease phenotypes has previously been observed in comparisons of several inbred mouse strains. Diverse mice have been utilized for decades in phenotypic and genetic analyses of candidiasis (29, 30). Three strains have also been used to assess acquired immunity to coccidioidomycosis, which demonstrated intermediate DBA primary susceptibility but resistance relative to BL/6 and outbred Swiss Webster mice (31). Those investigators identified a moderate DBA deficit in acquired immunity, where vaccinated mice failed to control pulmonary infection but suffered no fungal dissemination (31). A crossed BL/6 + DBA diversity outbred strain (B6D2F1/J) has been shown to tolerate *Coccidioides* infections where mice develop nonlethal chronic disease, which models human disease better than more susceptible mouse strains (32). To date, blastomycosis susceptibility across multiple, diverse strains has not been extensively studied. Susceptibility to primary infection was studied in several related (*M. musculus domesticus*-derived) strains 40 years ago, where DBA/2 mice were shown to have higher mortality rates than BL/6 mice (33). More recently, BALB/c and CD-1 mice were directly compared in yeast killing and cytokine production after B. dermatitidis infection (34).

Mouse genotype may influence host biology in ways that indirectly impact immunity. Two of the strains that were highly susceptible to primary infection included the NOD and NZO strains, where the males develop diabetes. Among NOD mice, 90% of females and 52% of males became diabetic by 30 weeks; the median incidence for females was 18 weeks (35). Thus, while our NOD mice were likely not diabetic when we studied them at 8 to 12 weeks, they may have been affected subclinically by metabolic changes, influencing their phenotype. For NZO mice, males and females of the NZO/Hl substrain exhibited impaired glucose tolerance (IGT), but subsequent type 2 maturity-onset diabetes (non-insulin-dependent diabetes mellitus [NIDDM]) development was limited to males, with a phenotype penetrance of 50% or lower. Thus, half of the NZO males were potentially diabetic, which likewise may have influenced their phenotype.

A strong interaction was observed between IFN-γ^+^ CD4 T cells and vaccination in CAST mice. In contrast, there was no such interaction for IL-17-producing CD4 T cells in this strain of mouse. We interpret this to mean that the vaccine effect, e.g., acquired resistance, is especially dependent on Th1 cells in this genetic background. Deeper insight into the genes that underpin and regulate this relationship could be divined further with CC mice. CC strains incorporate naturally varied alleles from each founder via a structured, randomized, eight-way breeding funnel before subsequent inbreeding into individual, fixed “genetic mosaic” strains. The DO panel is an outbred reference panel developed from the same founders as the CC and maintained through random breeding pairs (28). Through successive breeding generations, the DO panel gains recombination events that increase the mapping resolution and number of unique allelic combinations (28). Although lacking the reproducibility of the CC, the DO panel offers complex genetic heterogeneity that better models human genetics. When used in tandem, the CC and DO panels can complement and validate each other (36).

In this study, we have also developed robust flow cytometry-based immunophenotyping tools for diverse mice via thorough antibody validation. We identified antibodies that stain all MHCII variants in the founder strains, allowing us to identify relevant antigen-presenting cell populations. Previous flow cytometry-based phenotyping efforts of such populations in the founder strain mice have bypassed gating on this definitive marker and identified the variable dendritic cell subset markers alone (5, 6). Our efforts significantly improve diverse mouse immunophenotyping techniques, allowing a more thorough future analysis of immune responses in genetic reference panels. Our phenotypic description establishes a basis for the future use of genetic reference panels in identifying the genetic loci responsible for the acquisition of immunity to fungal and other microbial infections.

Although we identify broad variation across all nine investigated strains, we specifically describe primary susceptibility to infection paired with a deficit in acquired resistance in the DBA mouse strain. BL/6 and DBA mice tolerate primary infection similarly, yet the DBA mice fail to acquire vaccine resistance, which makes the direct comparison of BL/6 and DBA strains a potential model for acquired immunity. An RI genetic reference panel exists, derived from crossing BL/6 and DBA mice, and is called the BXD collection. The mouse genetic diversity represented in this panel is estimated to be only 16% due to the shared subspecies of origin (*M. musculus domesticus*). Despite a smaller reference gene pool, BXD mice nevertheless contain sufficient genetic diversity to identify novel candidate genes in multiple disease contexts (9, 37, 38) and can be used to understand genetic susceptibility to blastomycosis. The use of the BXD panel is more feasible than the use of the CC collection due to the smaller experimental scale and the greater availability of BXD strains. We can now use the BXD panel to investigate genetic sources of variation in susceptibility to both innate and especially acquired resistance during pulmonary infection with the human fungal pathogen B. dermatitidis.

## MATERIALS AND METHODS

### Fungi, mice, vaccination, and infection.

Blastomyces dermatitidis strains used were ATCC 26199, a wild-type virulent strain, and the isogenic, attenuated mutant lacking BAD1, designated strain 55 (39). Isolates of B. dermatitidis were maintained as yeast on Middlebrook 7H10 agar with oleic acid-albumin complex (Sigma Chemical Co., St. Louis, MO) at 37°C.

All strains of mice were obtained from the Jackson Laboratory and bred at the specific-pathogen-free BRMS Mouse Breeding Core. Mice were 8 to 12 weeks old at the initiation of the experiments. Mice were housed and cared for according to guidelines of the University of Wisconsin Animal Care Committee. All experiments were conducted per IACUC-approved protocol M005891-A02.

Mice were vaccinated subcutaneously (s.c.) with 10^5^ live cells of B. dermatitidis attenuated yeast strain 55 and rested for 14 days before a boost with 10^5^ live strain 55 yeast cells. Mice were challenged intratracheally at 14 days postboost with 2 × 10^4^ virulent ATCC 26199 B. dermatitidis yeast cells and analyzed for lung T cell responses and lung CFU at day 4 p.i. Weight change was assessed by weighing mice at the time of challenge and at the time of euthanasia and calculating the percent change in weight. Fungal burdens were measured from homogenized tissue by serial dilutions on brain heart infusion agar plates with penicillin and streptomycin. Zero colony counts are set to the detection limit (20).

### Tissue processing and cell culture.

Lungs were harvested and tissue was dissociated in Miltenyi MACS tubes with collagenase (1 mg/mL) and DNase (1 μg/mL) for 25 min at 37°C. Digested lungs were resuspended in 5 mL of 40% Percoll in RPMI 1640, and 3 mL of 66% Percoll in phosphate-buffered saline (PBS) was then underlaid (catalog number 17-0891-01; GE Healthcare). Samples were spun for 20 min at 2,000 rpm at room temperature. Lymphocytes were then harvested from the buffy coat layer and resuspended in complete RPMI 1640.

### T cell stimulation and flow cytometry.

Cell aliquots were incubated at 37°C for 5 h with 1 μg anti-CD28 (catalog number 553294; BD) and 0.1 μg anti-CD3 (catalog number 553057; BD) for *ex vivo* T cell stimulation. After 1 h of incubation, GolgiStop (catalog number 554724; BD) was added to samples to accumulate intracellular cytokines.

Samples for both surface staining and intracellular cytokine staining (ICS) were stained with a Live/Dead fixable near-infrared (IR) dead cell stain kit (catalog number L34975; Invitrogen) and Fc block (catalog number 553142; BD) for 10 min at room temperature. All antibodies were used at a 1:100 dilution. Cells were then stained in fluorescence-activated cell sorter (FACS) buffer (PBS plus 0.5% bovine serum albumin [BSA] and 2 mM EDTA) with surface antigens for 25 min at 4°C. ICS samples were then fixed and permeabilized (catalog number 554714; BD), followed by antibody staining for intracellular targets for 25 min at 4°C. All samples were washed and then fixed in 2% paraformaldehyde (PFA). Surface samples received 50 μL of AccuCheck counting beads (catalog number PCB100; Invitrogen) to determine absolute cell counts. Samples were acquired on a BD LSRFortessa instrument at the University of Wisconsin Carbone Cancer Center Flow Lab and analyzed using FlowJo X. The flow plots presented are for representative samples unless otherwise specified. For antibody clones and manufacturers, see Fig. 3. Multiplexing antibodies were used to simultaneously (in a single sample) identify multiple populations of leukocytes and lymphocytes with 12-color flow cytometry as previously described (22).

### Survival.

Vaccinated and unvaccinated BL/6 and DBA mice were challenged with 2 × 10^4^ cells of B. dermatitidis yeast strain ATCC 26199. Mice were assessed daily, and individual mice were euthanized when moribund and recorded as deceased. Surviving mice were euthanized at day 30 p.i., and lungs were collected for CFU analysis.

### Data analysis and visualization.

Statistics were calculated in R (version 3.6.3) using RStudio (version 1.2.5033). CFU data were log_10_ transformed and assessed for normalcy using skewness and kurtosis from the e1071 package (version 1.7-3). Regression analysis was conducted using multivariate type III analyses of variance (ANOVAs) (from the car package [version 3.0-11]). Contrasts for categorical variables were set to sum. A *P* value of <0.05 was considered statistically significant (*, *P* < 0.05; **, *P* < 0.005; ***, *P* < 0.001; ****, *P* < 0.0001). Groupwise comparisons were made using Tukey’s honestly significant difference (HSD) test of single and multivariate models.

Box, bar, and interaction plots were created using the R package ggplot2 (version 3.3.0). For box plots (Fig. 1A, Fig. 2A and B, Fig. 3B, and Fig. 5A and B), boxes represent the interquartile ranges, whiskers represent minima and maxima, and individual points are plotted. For bar plots (Fig. 1B, Fig. 3C and D, and Fig. 4B), bar heights represent group means, whiskers indicate 1 standard deviation, and individual points are plotted. For interaction plots (Fig. 4B), apices represent the means for a group. Survival analysis (Fig. 5C) was conducted in Prism 7 for Mac OS X, version 7.0d. Differences were determined by the Mantel-Cox rank test.
