# Supplementary figures and images for: Variation in Host Resistance to Blastomyces dermatitidis: Potential Use of Genetic Reference Panels and Advances in Immunophenotyping of Diverse Mouse Strains
Source: mBio. 2022 Jan 4;13(1):e03400-21. doi: 10.1128/mbio.03400-21 (PMC8725596; doi:10.1128/mbio.03400-21)

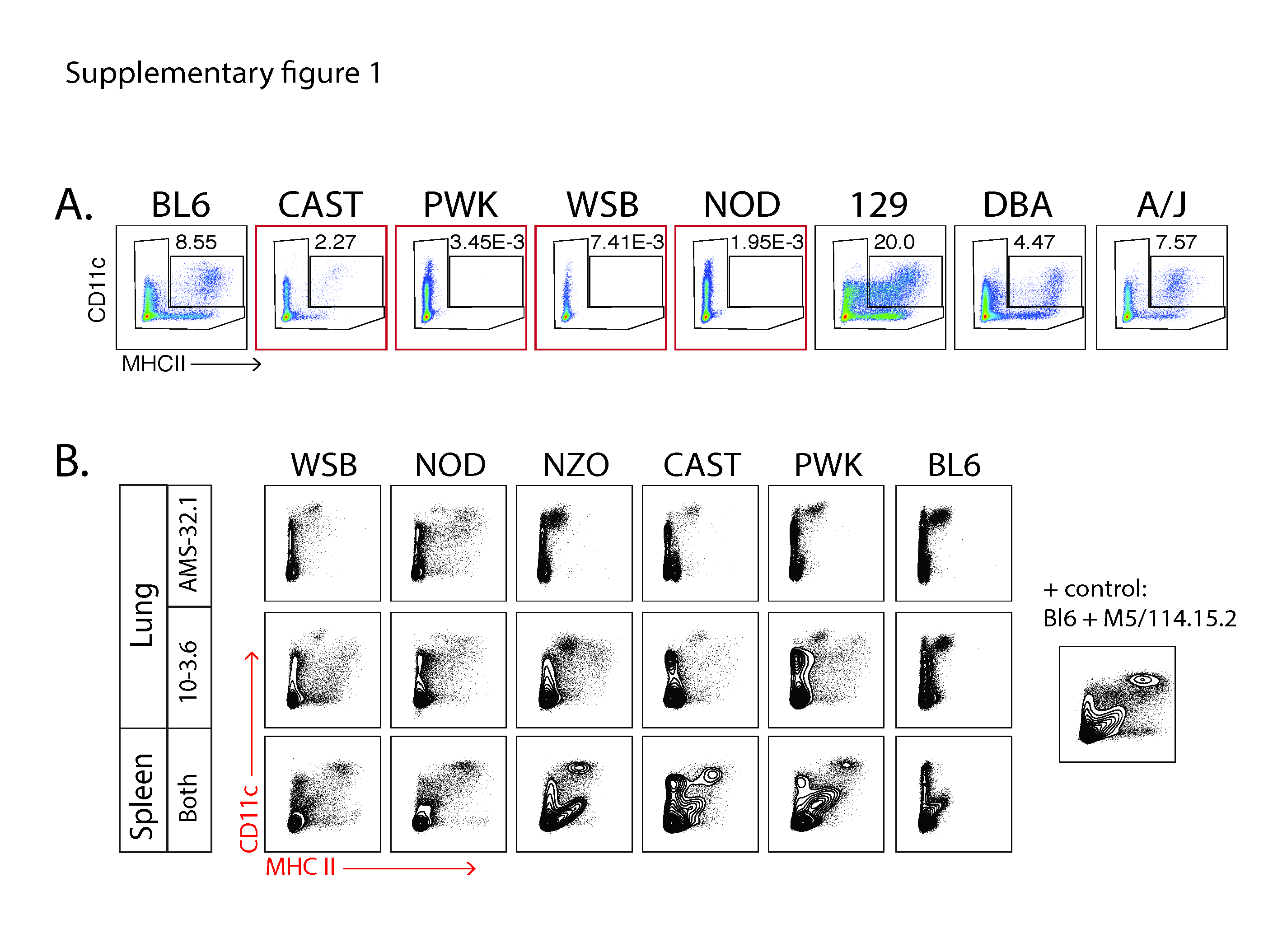

Supplement: FIG S1 [file mbio.03400-21-sf001.tif]

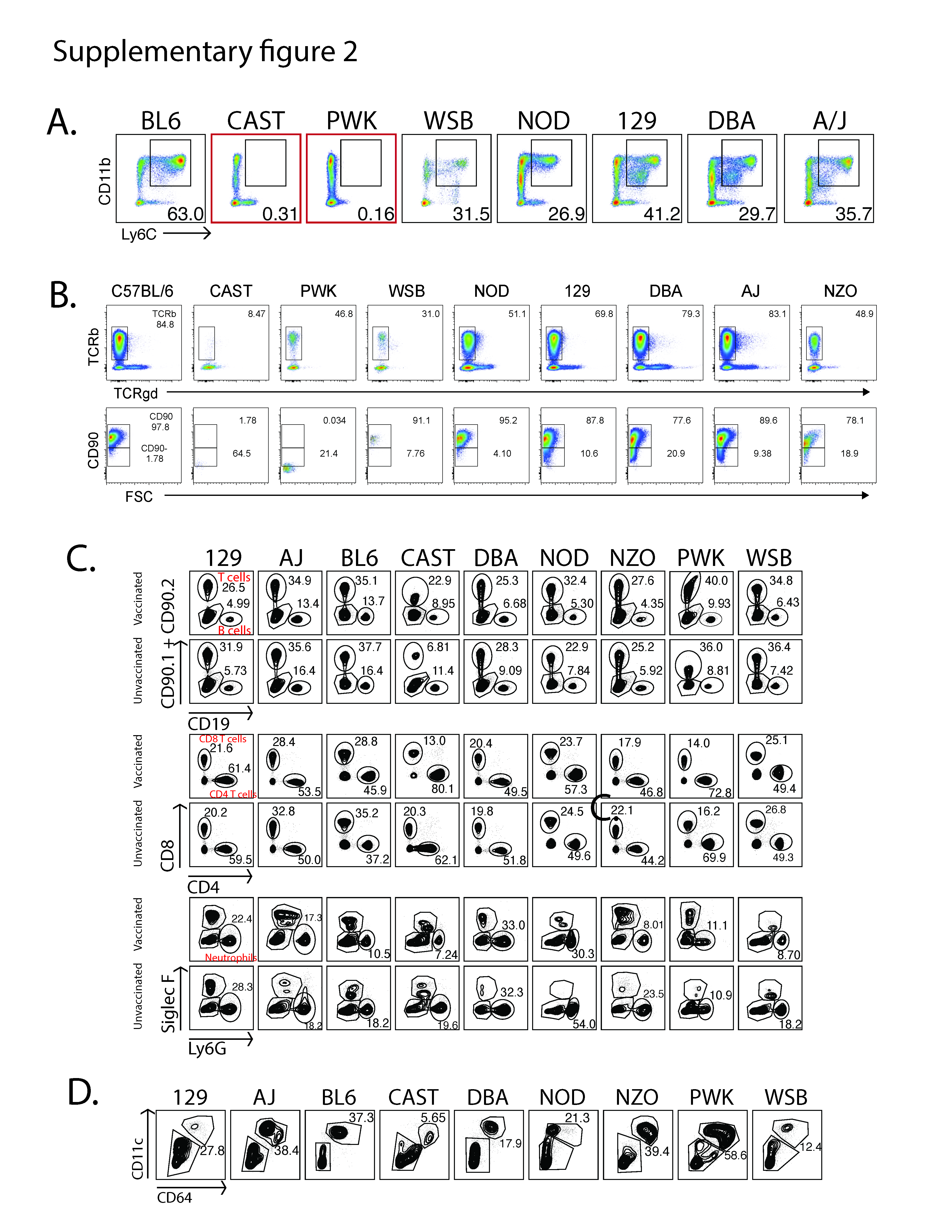

Supplement: FIG S2 [file mbio.03400-21-sf002.tif]

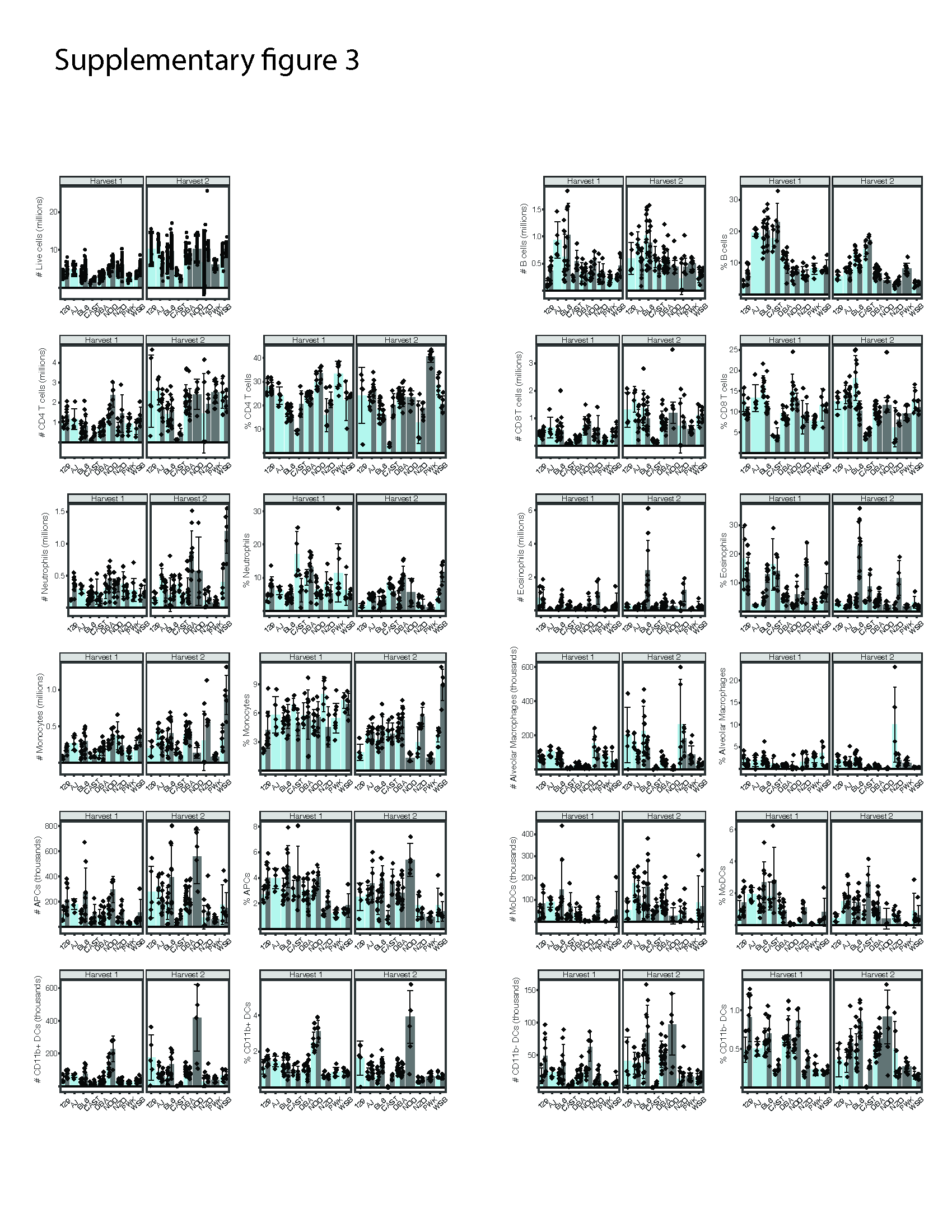

Supplement: FIG S3 [file mbio.03400-21-sf003.tif]

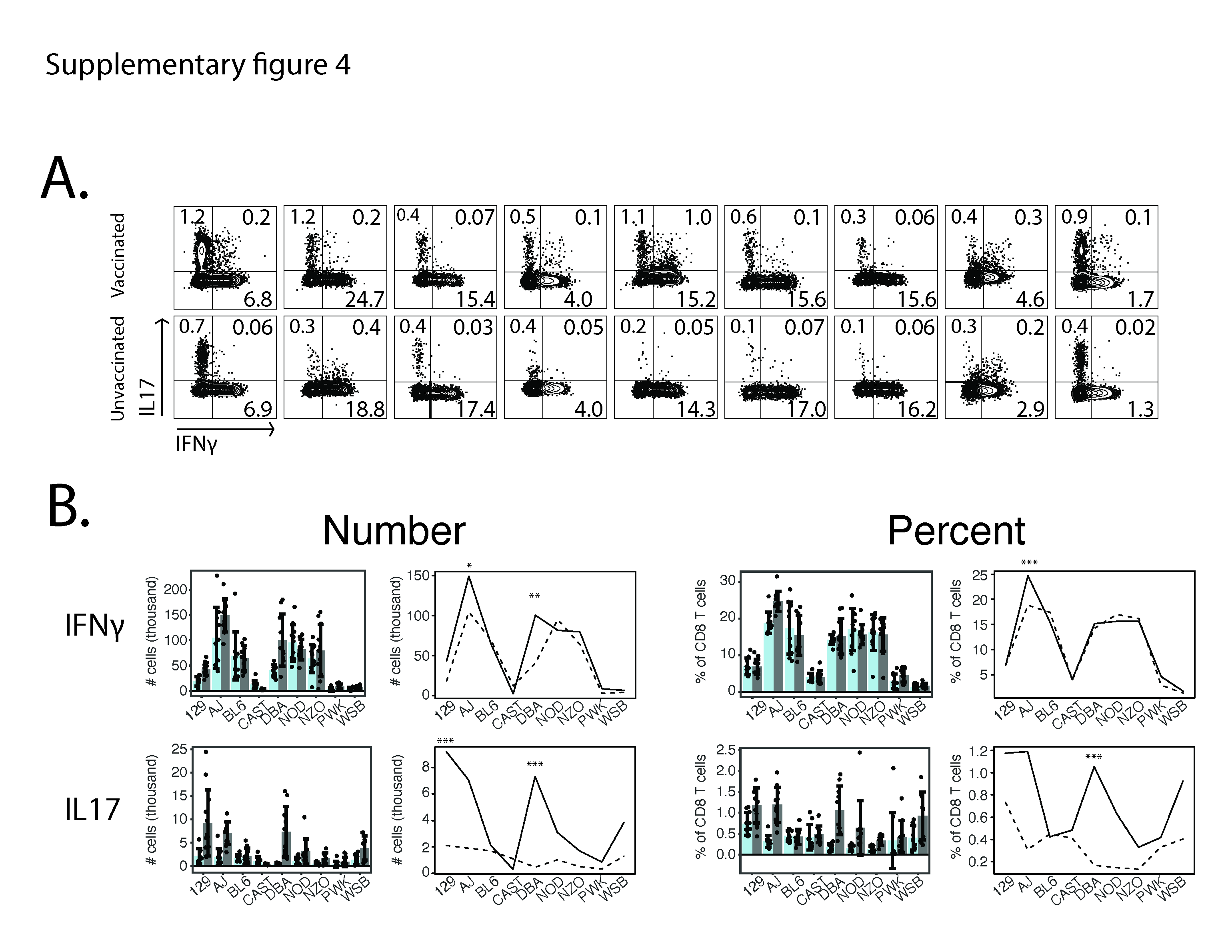

Supplement: FIG S4 [file mbio.03400-21-sf004.tif]
